# Non-Celiac Gluten Sensitivity: The New Frontier of Gluten Related Disorders

**DOI:** 10.3390/nu5103839

**Published:** 2013-09-26

**Authors:** Carlo Catassi, Julio C. Bai, Bruno Bonaz, Gerd Bouma, Antonio Calabrò, Antonio Carroccio, Gemma Castillejo, Carolina Ciacci, Fernanda Cristofori, Jernej Dolinsek, Ruggiero Francavilla, Luca Elli, Peter Green, Wolfgang Holtmeier, Peter Koehler, Sibylle Koletzko, Christof Meinhold, David Sanders, Michael Schumann, Detlef Schuppan, Reiner Ullrich, Andreas Vécsei, Umberto Volta, Victor Zevallos, Anna Sapone, Alessio Fasano

**Affiliations:** 1 Department of Pediatrics, Università Politecnica delle Marche, Ancona 60121, Italy; E-Mail: catassi@tin.it; 2 Departamento de Medicina, Hospital de Gastroenterología “Dr. Carlos Bonorino Udaondo”, Buenos Aires 1264, Argentina; E-Mail: jbai@intramed.net; 3 Department of Gastroenterology and Liver Diseases, CHU Grenoble 38043, France; E-Mail: bbonaz@chu-grenoble.fr; 4 Department of Gastroenterology and Hepatology, Vrije Universiteit Medical Center, Amsterdam 1081 HV, The Netherlands; E-Mail: g.bouma@vumc.nl; 5 Gastroenterology Unit, Department of Experimental and Clinical Biomedical Sciences, University of Florence, Florence 50134, Italy; E-Mail: a.calabro@dfc.unifi.it; 6 Department of Internal Medicine, “Giovanni Paolo II” Hospital, Sciacca (AG) and University of Palermo, Sciacca 92019, Italy; E-Mail: acarroccio@hotmail.com; 7 Pediatric Gastroenterology Unit, Hospital Universitari de Sant Joan de Reus, Universitat Rovira i Virgili, Tarragona 43204, Spain; E-Mail: gcv@tinet.cat; 8 Department of Medicine and Surgery, University of Salerno, Baronissi Campus, Salerno 84081, Italy; E-Mail: cciacci@unisa.it; 9 Interdisciplinary Department of Medicine, University of Bari, Bari 70124, Italy; E-Mails: fernandacristofori@gmail.com (F.C.); rfrancavilla@gmail.com (R.F.); 10 Gastroenterology Unit, Department of Pediatrics, University Medical Centre Maribor, Maribor 2000, Slovenia; E-Mail: jernej.dolinsek@ukc-mb.si; 11 Centro Prevenzione e Diagnosi Malattia Celiaca Fondazione IRCCS Ca Granda, Milan 20122, Italy; E-Mail: lucelli@yahoo.com; 12 Department of Medicine, Celiac Disease Center, Columbia University Medical Center, New York, NY 10032, USA; E-Mail: pg11@columbia.edu; 13 Division of Gastroenterology and Internal Medicine, Hospital Porz am Rhein, Köln 51149, Germany; E-Mail: w.holtmeier@khporz.de; 14 German Research Center for Food Chemistry, Leibniz Institute, Freising 85354, Germany; E-Mail: peter.koehler@tum.de; 15 Division of Pediatric Gastroenterology and Hepatology, Dr. von Hauner Children’s Hospital, University of Munich Medical Center, Munich 80337, Germany; E-Mail: sybille.koletzko@med.uni-muenchen.de; 16 Practice of Nutrition Therapy Meinhold & Team, Köln 50674, Germany; E-Mail: praxis@christof-meinhold.de; 17 Department of Gastroenterology and Hepatology, Royal Hallamshire Hospital and University of Sheffield Medical School, Sheffield S10 2JF, UK; E-Mail: david.sanders@sth.nhs.uk; 18 Department of Gastroenterology, Rheumatology and Infectiology, Charité University Medicine, Berlin 10203, Germany; E-Mails: michael.schumann@charite.de (M.S.); reiner.ullrich@charite.de (R.U.); 19 Department of Medicine I, University Medical Center, Johannes Gutenberg University Mainz, Mainz 55131, Germany; E-Mails: dschuppa@bidmc.harvard.edu (D.S.); zevallos@uni-mainz.de (V.Z.); 20 Division of Gastroenterology and Celiac Center, Beth Israel Deaconess Medical Center and Harvard Medical School, Boston, MA 02215, USA; 21 St. Anna Children’s Hospital, Vienna 1090, Austria; E-Mail: andreas.vecsei@stanna.at; 22 Department of Medical and Surgical Sciences, University of Bologna, Bologna 40138, Italy; E-Mail: uvolt@yahoo.com; 23 Department of Gastroenterology, Second University of Naples, Naples 80136, Italy; E-Mail: annasapone@yahoo.it; 24 Pediatric Gastroenterology and Nutrition, MassGeneral Hospital for Children, Boston, MA 02129, USA

**Keywords:** gluten sensitivity, celiac disease, wheat allergy, gluten-related disorders, gluten-free diet

## Abstract

Non Celiac Gluten sensitivity (NCGS) was originally described in the 1980s and recently a “re-discovered” disorder characterized by intestinal and extra-intestinal symptoms related to the ingestion of gluten-containing food, in subjects that are not affected with either celiac disease (CD) or wheat allergy (WA). Although NCGS frequency is still unclear, epidemiological data have been generated that can help establishing the magnitude of the problem. Clinical studies further defined the identity of NCGS and its implications in human disease. An overlap between the irritable bowel syndrome (IBS) and NCGS has been detected, requiring even more stringent diagnostic criteria. Several studies suggested a relationship between NCGS and neuropsychiatric disorders, particularly autism and schizophrenia. The first case reports of NCGS in children have been described. Lack of biomarkers is still a major limitation of clinical studies, making it difficult to differentiate NCGS from other gluten related disorders. Recent studies raised the possibility that, beside gluten, wheat amylase-trypsin inhibitors and low-fermentable, poorly-absorbed, short-chain carbohydrates can contribute to symptoms (at least those related to IBS) experienced by NCGS patients. In this paper we report the major advances and current trends on NCGS.

## 1. Introduction

Gluten sensitivity (GS) was originally described in the 1980s [1] and a recently “re-discovered” syndrome entity, characterized by intestinal and extra-intestinal symptoms related to the ingestion of gluten-containing food, in subjects that are not affected with either celiac disease (CD) or wheat allergy (WA). Following the landmark work by Sapone and coworkers, describing the clinical and diagnostic features of GS in the year 2010 [2], a rapidly increasing number of papers have been published by many independent groups, confirming that GS should definitely be included in the spectrum of gluten-related disorders. However, many aspects of GS epidemiology, pathophysiology, clinical spectrum, and treatment are still unclear. Given the recent increase of the gluten-free market worldwide, partially sustained by individuals who claim a medical necessity to undertake a gluten-free diet (GFD), there is a need of “separating the wheat from the chaff” [3]. This goal will be achieved by (a) proper scientific information, (b) shared definitions, and (c) prospective, multi-center studies addressing the many unsolved issues on GS. In order to develop a consensus on new nomenclature and classification of gluten-related disorders, a panel of experts first met in London, in February 2011. The panel proposed a series of definitions and developed a diagnostic algorithm that has been recently published [4].

After the 2011 London Meeting, many new papers have been published on GS. Although its frequency in the general population is still unclear, epidemiological data have been generated that can help establish the magnitude of the problem. Clinical studies further defined the identity of GS and its possible implications in human disease. An overlap between the irritable bowel syndrome (IBS) and GS has been suspected, requiring even more stringent diagnostic criteria. The first case reports of GS in children have been described. Lack of biomarkers is still a major limitation of clinical studies, making the differential diagnosis with other gluten related disorders, as well conditions independent to gluten exposure, difficult.

Evaluation and discussion of this new information was the aim of a Second Expert Meeting on GS that was held in Munich, November 30–December 2, 2012. In this paper we report the major advances and current trends on GS, as presented and debated at the Munich meeting.

## 2. Nomenclature

At least three papers have recently addressed the issue of defining gluten-related disorders [4,5,6]. Interestingly, one of these [4] ranks among the most frequently downloaded paper of the publishing journal (*BMC Medicine*), particularly by physicians, internists or general pediatricians, and directors of diagnostic labs. There is a general agreement that the term “gluten-related disorders” is the umbrella-term to be used for describing all conditions related to ingestion of gluten-containing food. CD is a chronic small intestinal, immune-mediated, enteropathy precipitated by exposure to dietary gluten and related prolamines in genetically predisposed individuals, characterized by specific autoantibodies against tissue transglutaminase 2 (anti-TG2) and endomysium (EMA). WA is an adverse immunologic reaction to wheat proteins. In the pathogenesis of WA, wheat specific IgE antibodies play a central role, however non-IgE-mediated WA does exist [7], and this form may be difficult to distinguish from GS.

GS, which this review will focus on primarily, is a condition in which symptoms are triggered by gluten ingestion, in the absence of celiac-specific antibodies and of classical celiac villous atrophy, with variable Human Leukocyte Antigen (HLA) status and variable presence of first generation anti-gliadin antibodies (AGA). The “labeling” of this disorder was a matter of debate among the panel experts. In order to avoid confusion with CD, sometimes defined as gluten-sensitive enteropathy, “non celiac gluten sensitivity” (NCGS) appeared as an improved definition. Doubtless this is still too vague a terminology, simply reflecting the poor knowledge of the pathophysiology of this condition. As triggering cereal proteins could include fractions other than gluten (see Section 10 below) some panelists were in favor of “non-celiac wheat (protein) sensitivity”, a terminology that would however conflict with the possibility that other gluten-containing cereals (rye, barley) may be offensive for the “gluten sensitive” patient. Bearing these limitations in mind, the experts’ panel agreed that this entity can provisionally be defined as NCGS, a definition requiring refinement in the future.

## 3. Epidemiology

The overall prevalence of NCGS in the general population is still unknown, mainly because many patients are currently self-diagnosed and start a GFD without medical advice or consultation. However, new data confirm that this is not an uncommon disorder at all. In a region of New Zealand, 5% of children reported non-CD-related avoidance of gluten-containing food [8]. Gluten avoidance was associated with improvement of nonspecific behavioral and gastrointestinal complaints [9]. It remains to be elucidated how many children reporting gluten avoidance were indeed affected by NCGS, as the vast majority of the children involved in this study were not tested for CD nor underwent to an intestinal biopsy. In a US study performed on 7762 unselected persons aged six years or older who participated in the National Health and Nutrition Examination Survey (NHANES) 2009–2010, Digiacomo *et al*. found a 0.55% prevalence of persons on a self-reported GFD. The prevalence was higher in females and older participants [10]. Many of the NHANES subjects on a GFD could indeed be affected by NCGS, however this is likely to be an underestimate as (a) the possible relationship between gastro-intestinal symptoms and gluten intake was not systematically explored in this population sample, and (b) the NHANES survey was conducted before NCGS was described in the medical literature.

The analysis of the epidemiology of IBS provides an indirect estimate of intestinal NCGS frequency. According to recent population-based surveys performed in Northern Europe, the prevalence of IBS in the general adult population is 16%–25% [11,12]. In a selected (and, therefore, probably biased) series of adults with IBS, the frequency of NCGS, documented by a double-blind, placebo-controlled challenge, was 28% [13]. In the large study performed by Carroccio *et al*., 276 out of 920 (30%) subjects with IBS-like symptoms, according to the Rome II criteria, suffered from wheat sensitivity or multiple food hypersensitivity, including wheat sensitivity [14]. Should a consistent proportion of IBS patients be affected with NCGS, the prevalence of NCGS in the general population could well be higher than CD (1%).

Although risk factors for NCGS have not yet been identified, the disorder seems to be more common in females and in young/middle age adults. The prevalence of NCGS in children is still unknown.

## 4. Clinical Picture and Natural History

NCGS is characterized by symptoms that usually occur soon after gluten ingestion, disappear with gluten withdrawal and relapse following gluten challenge, within hours or few days. The “classical” presentation of NCGS is a combination of IBS-like symptoms, including abdominal pain, bloating, bowel habit abnormalities (either diarrhea or constipation), and systemic manifestations such as “foggy mind”, headache, fatigue, joint and muscle pain, leg or arm numbness, dermatitis (eczema or skin rash), depression, and anemia [2,15]. When seen at the specialty clinic, many NCGS patients already report the causal relationship between the ingestion of gluten-containing food and worsening of symptoms. In children, NCGS manifests with typical gastrointestinal symptoms, such as abdominal pain and chronic diarrhea, while the extra-intestinal manifestations seem to be less frequent, the most common extra-intestinal symptom being tiredness [16].

During the last decade, several studies suggested a relationship between NCGS and neuropsychiatric disorders (see following paragraphs).

While it is undisputable that in some cases the positive effect of gluten withdrawal can be explained by a placebo effect, this is not the case in true NCGS. In a double-blind randomized placebo-controlled study design, Biesiekierski *et al*. found that IBS-like symptoms of NCGS were more frequent in the gluten-treated group (68%) than in subjects on placebo (40%) [13]. Furthermore a recent study found no significant differences between CD and NCGS patients regarding personality traits, level of somatization, quality of life, anxiety, and depressive symptoms. The somatization level was low in both diseases. Additionally, symptom increase after a gluten challenge was not related to personality in NCGS patients [17].

No major complication of untreated NCGS has so far been described; especially autoimmune comorbidity, as observed in CD, has not been reported so far. However, natural history data on NCGS are still lacking. Therefore it is difficult to draw firm conclusions on the outcome of this condition.

## 5. NCGS and IBS: A Complex Relationship

The complex relationship between IBS and dietary proteins has been recently reviewed [18]. Patients with CD often report symptoms compatible with IBS persisting after treatment with the GFD. In a recent meta-analysis the pooled prevalence of IBS-type symptoms in patients with treated CD was 38.0% (95% CI, 27.0%–50.0%). The pooled odds ratio (OR) for IBS-type symptoms was higher in patients with CD than in controls (5.60; 95% CI, 3.23–9.70). In patients who were non-adherent with a GFD, the pooled OR for IBS-like symptoms, compared with those who were strictly adherent, was 2.69 (95% CI, 0.75–9.56) [19].

That gluten ingestion may elicit gastrointestinal symptoms in non-CD patients has recently been shown in subjects affected with the D variant (diarrhea-predominant) of IBS, by Vazquez-Roque and coworkers. Subjects on a gluten containing diet (GCD) had more bowel movements per day, particularly those with HLA-DQ2 and/or DQ8 genotypes. The GCD was associated with higher small bowel permeability. Patients on the GCD had a small decrease in expression of zonula occludens 1 in small bowel mucosa, and significant decreases in expression of zonula occludens 1, claudin-1, and occludin in rectosigmoid mucosa; again the effects of the GCD on expression were significantly greater in HLA-DQ2/8–positive patients. On the other hand, the GCD *vs.* the GFD had no significant effects on gastrointestinal transit or histology. It was concluded that gluten alters bowel barrier functions in patients with IBS-D, particularly in HLA-DQ2/8–positive patients. These data provided mechanistic explanations for the observation that gluten withdrawal may improve patient symptoms in IBS [20].

How specific the effect of gluten withdrawal from the diet of patients with IBS is, still remains to be elucidated. Besides gluten, wheat, and wheat derivatives contain other constituents that could play a role in triggering symptoms in IBS patients, e.g., amylase-trypsin inhibitors (ATIs, see below) and fructans. In a second study, Biesiekirski *et al*. reported on 37 patients with IBS/self-reported NCGS investigated by a double-blind crossover trial. Patients were randomly assigned to a period of reduced low-fermentable, poorly-absorbed, short-chain carbohydrates (fermentable oligo-, di-, and mono-saccharides and polyols = FODMAPs) diet and then placed on either a gluten or whey proteins challenge. In all participants, gastrointestinal complaints consistently improved during reduced FODMAP intake, but significantly worsened to a similar degree when their diets included gluten or whey proteins [21].FODMAPS list includes fructans, galactans, fructose, and polyols that are contained in several foodstuffs, including wheat, vegetables, and milk derivatives. These results raise the possibility that the positive effect of the GFD in patients with IBS is an unspecific consequence of reducing FODMAPs intake, given that wheat is one of the possible sources of FODMAPs. However, it should be stressed that FODMAPs cannot be entirely and exclusively responsible for the symptoms experienced by NCGS subjects, since these patients experience a resolution of symptoms while on a GFD despite continuing to ingest FODMAPs from other sources, like legumes (a much richer source of FODMPs than wheat). Nevertheless, based on the results reported by Biesiekirski *et al*. is also possible that there are IBS cases entirely due to FODMAPs that, therefore, cannot be classified as affected by NCGS [21].

## 6. Is Autism Part of the NCGS Spectrum?

Autism Spectrum Disorders (ASD) are chronic behavioral conditions, with onset before three years of age. ASD are one of the fastest growing developmental disabilities in the United States. They present with a wide range of stereotyped, repetitive behaviors, social and language impairment. Function and outcome is affected not only by core deficits but also by associated behaviors such as hyperactivity, aggression, anxiety, and depression. Many studies have indicated that behavioral therapy and medication may be at least partially helpful in the management of children with ASD. Research on the effect of diet and nutrition on autism has been increasing in the past two decades, particularly on the symptoms of hyperactivity and attention. One of the most popular interventions for ASD is the gluten free casein free (GFCF) diet.

The possible effect of the GFCF in children with autism is not due to underlying CD, since an association between these two conditions has never been clearly confirmed by serological screening studies [22]. It has been hypothesized that some symptoms may be caused by opioid peptides formed from the incomplete breakdown of foods containing gluten and casein. Increased intestinal permeability, also referred to as the “leaky gut syndrome,” has been suspected in ASD to be part of the chain of events that allows these peptides to cross the intestinal membrane, enter the bloodstream, and cross the blood-brain barrier, affecting the endogenous opiate system and neurotransmission within the nervous system. The resulting excess of opioids is thought to lead to behaviors noted in ASD, and the removal of these substances from the diet could determine a change in autistic behaviors [23]. The leaky gut/autism connection has fuelled a strong debate within the scientific community, far from being settled. A recent study has reported a high percentage of abnormal intestinal permeability test (as established by the lactulose/mannitol ratio) among patients with autism (36.7%) and their relatives (21.2%) compared with normal subjects (4.8%). Patients with autism on a reported GFCF diet had significantly lower intestinal permeability test values compared with those who were on an unrestricted diet and controls [24]. However, the degree of correlation between abnormal intestinal permeability to sugars (lactulose and mannitol) and proteins/peptides remains to be established. It should also be pointed out that, in a pilot study, Robertson *et al*. did not detect any changes in intestinal permeability in a small cohort of ASD children [25]. The finding of IgG class antibodies directed against food antigens is considered indirect evidence of increased intestinal permeability. Children with autism have significantly higher levels of IgG antibody (but not IgA) to gliadin compared with healthy controls, particularly in those with gastrointestinal symptoms [26]. Recent studies confirmed these findings and also reported an increase in antibodies directed to several other food allergens, including casein and whole milk [27].

Despite its popularity, the efficacy of the GFCF diet in improving autistic behavior remains not conclusively proven. A 2008 Cochrane review reported that only two small RCTs investigated the effect of GFCF diet in children with ASD (*n* = 35). There were only three significant treatment effects in favor of the diet intervention: overall autistic traits, mean difference (MD) = −5.60; social isolation, MD = −3.20 and overall ability to communicate and interact, MD = 1.70. In addition three outcomes were not different between the treatment and control group while differences for ten outcomes could not be analyzed because data were skewed. The review concluded that the evidence for efficacy of these diets is poor, and large scale, good quality randomized controlled trials are needed [28].

By using a two-stage, randomized, controlled study of GFCF diet of children with ASD, Whiteley and coworkers recently reported significant group improvements in core autistic and related behaviors after eight and 12 months on diet. The results showed a less dramatic change between children having been on diet for eight and children in diet for 24 months, possibly reflective of a plateau effect [29].

The above data suggest that removing gluten from the diet may positively affect the clinical outcome in some children diagnosed with ASD, indicating that autism may be part of the spectrum of NCGS, at least in some cases. However, a word of caution is necessary to stress the fact that only a small, selected sub-group of children affected by ASD may benefit from an elimination diet. Additional investigations are required in order to identify phenotypes based on best- and non-response to dietary modifications and assess any biological correlates including anthropometry before considering a dietary intervention.

## 7. Gluten-Related Disorders and Schizophrenia

An association between schizophrenia and CD was noted in reports spanning back to the 1960s [30]. In 1986 a double-blind gluten-free/gluten-load controlled trial of 24 patients conducted by Vlissides *et al*. showed changes in symptom profile of schizophrenics in response to exclusion of gluten from the diet [31]. On the other hand, a small blind study conducted by Potkin *et al*. showed no differences in the clinical status of eight schizophrenic patients on a 5-week gluten challenge in an in-patient setting, as measured by the Brief Psychiatric Rating Scale [32]. A subsequent study by Storms *et al*. tested 26 schizophrenic patients on a locked ward assigned to either a gluten-free or high gluten diet. No differences were found between the groups on their performance in a battery of psychological tests [33]. A recent study using blood samples from the Clinical Antipsychotic Trials of Intervention Effectiveness (CATIE) found that 5.5% of the subjects with schizophrenia had a high level of anti-tTG antibodies (compared to 1.1% in the healthy control sample) and 23.1% had AGA IgG positivity compared with 3.1% in controls. Interestingly enough, a large proportion of tTG positive subjects resulted EMA negative, questioning the possibility that their tTG positivity was related to CD. Indeed, only 2% of schizophrenic patients fulfilled the CD diagnostic criteria (both anti-tTG and EMA positive), questioning the role of CD in schizophrenia [34]. Additional studies revealed that most of the tTG positive subjects were tTG-6 positive, suggesting that these antibodies are more a biomarker of neuro-inflammation than CD [35]. This study indicated the existence of a specific immune response to gluten in some of these patients, probably related to NCGS. Other studies confirmed the high prevalence of antibodies to AGA among people with schizophrenia [36], however the exact mechanism underlying the observed improvement of symptoms in some patients with the GFD has remained elusive. Immunological mechanisms have been proposed, including the assertion that a subgroup of schizophrenics suffer from food intolerances that benefit from the adoption of a GFD. The beneficial effect of a GFD may also be achieved via circulating food-derived peptides (exorphins) exerting an influence on physiological processes in the brain (same mechanism as described in the autism paragraph). If it were true that a subset of schizophrenic patients did exhibit symptoms due to sensitivity to gluten, then not only would treatment for these individuals be easier and more efficient than neuroleptics but also their quality of life would improve.

In summary, the role of NCGS in conditions affecting the nervous system remains a highly debated and controversial topic that requires additional, well-designed studies to establish the real role of gluten as a triggering factor in these diseases.

## 8. Laboratory Evaluation

So far no specific biomarker of NCGS has been identified. Recently, Volta and colleagues reported on the pattern of CD serology found in 78 untreated patients affected with NCGS. Many patients displayed an elevated prevalence of high titer, “first-generation” IgG AGA directed against native gliadin (56.4%). The prevalence of IgG AGA detected in NCGS, although lower than that found in CD (81.2%), was much higher than other pathologic conditions such as connective tissue disorders (9%) and autoimmune liver diseases (21.5%) as well as in the general population and healthy blood donors (2%–8%). On the other hand, the prevalence of IgA AGA in NCGS patients was very low (7.7%). Noteworthy, the “best” CD markers, namely IgG deamidated gliadin peptide (DGP) antibodies, IgA tTGA, and IgA EMA, were always negative in NCGS patients, except for an isolated positivity at a very low titer for IgG DGP. The consistent negativity for IgG DGP, whose synthesis “*in vivo*” is an expression of the interaction between tissue transglutaminase and gliadin peptides, seems to exclude the involvement of adaptive immunity in NCGS pathogenesis. Interestingly enough, ELISA activities of IgA tTGA in NCGS patients were very low with 30% of them displaying values < 1 AU (none of them had IgA deficiency) [15].

The CD-predisposing HLA-DQ2 and DQ8 genotypes are found in 50% of NCGS patients, a prevalence that is lower than CD (95%) and only slightly higher than the general population (30%) [4].

In the work of Sapone and coworkers all subjects (11 patients with NCGS, 13 with CD, and seven controls), underwent upper duodenal endoscopy for small intestinal biopsy. Those with NCGS revealed normal to mildly inflamed mucosa (Marsh 0 to 1), while all CD patients showed partial or subtotal villous atrophy with crypt hyperplasia. As expected, CD patients had increased numbers of CD3+ IELs (>50/100 enterocytes) compared to controls, while NCGS patients had a number of CD3+ IELs intermediate between CD patients and controls in the context of relatively conserved villus architecture. The numbers of TCR-γδ IELs were only elevated in CD subjects (>3.4/100 enterocytes), while in NCGS patients the numbers of γδ IELs were similar to those in controls [2]. Recently, activation of circulating basophils [14] and increased infiltration of duodenal lamina propria with eosinophils [37] have been described.

## 9. Diagnosis

NCGS diagnosis is sometimes suspected by the patients themselves based on food withdrawal and introduction. Physicians may then concur if there has been the exclusion of other forms of gluten-induced disease (CD and WA) by appropriate serological and/or biopsy tests. Specific IgE might normalize if the patients are already on GFD and this might be a potential pitfall in diagnosis of WA The finding that symptoms disappear after gluten elimination adds weight to the diagnosis of NCGS, which is definitely proven by a double-blind (or open) oral gluten challenge performed after at least three weeks of GFD.

Based on a combination of clinical, biological, genetic and histological data, it is possible to differentiate the three gluten-related conditions (WA, CD, and NCGS), using recently published algorithms [4]. Since there is some degree of overlap between NCGS and other forms of wheat-exclusion responsive conditions (e.g., IBS responsive to low FODMAPs diet, non-IgE mediated WA), periodical patient reassessment (e.g., every 6–12 months), including an accurate dietary interview, is strongly recommended.

## 10. Pathogenesis

The pathophysiology of NCGS is under scrutiny. In the study conducted by Sapone *et al*. [2], NCGS subjects showed a normal intestinal permeability and claudin-1 and ZO-1 expression compared with celiac patients, and a significantly higher expression of claudin-4. In the same NCGS patients, the up-regulation of claudin-4 was associated with an increased expression of toll-like receptor-2 and a significant reduction of T-regulatory cell marker FoxP3 relative to controls and CD patients. Additionally, an increase in IELs of the classes α and β, but no increase in adaptive immunity-related gut mucosal gene expression, including interleukin (IL)-6, IL-21, and interferon-γ (IFN-γ), was detected in NCGS. These changes suggested an important role of the intestinal innate immune system in NCGS, without any involvement of the adaptive immune response. In a study aimed at exploring and comparing the early mucosal immunological events in CD and NCGS, Brottveit *et al*. confirmed that CD patients mounted a concomitant innate and adaptive immune response to gluten challenge. NCGS patients only showed increased IFN-γ levels after gluten challenge and increased density of intraepithelial CD3(+) T cells at baseline [38]. These findings open the possibility of an adaptive component as well in the pathogenesis of NCGS.

The trigger/s of mucosal events leading to NCGS is not necessarily represented by the same array of gluten peptides responsible for CD development. Unlike the duodenal mucosa from patients with CD, upon incubation with gliadin, mucosa from patients with NCGS does not express markers of inflammation, and their basophils are not activated by gliadin [39]. *In vitro* studies suggest that wheat ATIs could play a major role as triggers of the innate immune response in intestinal monocytes, macrophages and dendritic cells eventually leading to NCGS. Wheat ATIs are a family of five or more homologous low-molecular-weight proteins highly resistant to intestinal proteolysis. They are known to be the major allergen responsible for baker’s asthma. ATIs engage the TLR4-MD2-CD14 complex and lead to up-regulation of maturation markers and elicit release of pro-inflammatory cytokines in cells from celiac and non-celiac patients and in celiac patients’ biopsies [40].

## 11. Current and Future Trends

The vast majority of celiac experts initially reacted with a great deal of skepticism to the concept of NCGS existence and the fact that it was a separate entity from CD. For those that witnessed the initial struggle of convincing health care professionals that CD was not confined within European boundaries this was a *déjà vu*. Indeed, we are now with NCGS where we probably were with CD forty years ago. In the 1980s we knew that CD existed, but we had little information on the mechanisms leading to the enteropathy, the genetic component of the disease, what kind of immune response was involved in the pathogenesis of the disease, its multifaceted clinical presentation, and its complication. We lacked robust screening tools to conduct well-design epidemiological studies and had little understanding on the most appropriate management of the disease and its complications. The confusion about NCGS stems from the few facts, and the many fantasies, currently available on this topic. The best testimonial of this concept is the comparison of the literature published on both conditions during the past 63 years. The publications on CD doubled every 20 years from approximately 2500 in the period of 1950–70 to ~9500 in the period 1991–2010, with already more than 2000 papers published between 2011 and 2013. Conversely, there were almost no scientific reports on NCGS before 1970 and only a handful number of papers have been published ever since, most of them after 2005. The increase interest in NCGS is testified by the decreased NCGS/CD publication ratio that dropped from 1:438 in the period 1950–70 to 1:10 in the period 2010–13 (Table 1).

**Table 1:** Trends in publication on celiac disease (CD) and non-celiac gluten sensitivity (NCGS) during the last decades.

Timeline	CD	NCGS	NCGS/CD ratio
1950–1970	2632	6	1:438
1971–1990	4915	118	1:43
1991–2010	9498	733	1:13
2011–2013	2014	188	1:10

Given the limited literature on the topic, it should not come as a surprise that there are still numerous questions about NCGS that should be addressed. Is NCGS permanent or transitory? Is the threshold of sensitivity the same for everybody, or change from subject to subject and in the same subject over time? How frequent is NCGS? The range reported in the literature is between 0.5% and 6%, based on poorly conducted studies and on definitions of the disease that varies widely from one report to another. Only recently, well-conducted studies based on double blind, placebo control design are providing evidence-based data on the prevalence of NCGS in specific clinical conditions, particularly IBS [13]. There is the strong need for more coordinated efforts to perform large multicenter studies for those conditions, including autism and schizophrenia, in which NCGS has been indicated as a possible cause in a subgroup of these patients. The lack of validated biomarkers for a diagnosis not based on exclusion criteria is judged to be of paramount importance by many experts in the field. Currently a large multicenter placebo-controlled study is underway to achieve this goal and, hopefully, will provide tools for a more correct diagnosis and for more rigorous studies to establish the prevalence of NCGS in specific conditions and in the general population. Recent studies raised the possibility that, beside gluten [13] and wheat ATIs [40], low-fermentable, poorly-absorbed, short-chain carbohydrates [21] can contribute to symptoms (at least those related to IBS) experienced by NCGS patients. These new findings need corroboration through additional studies involving larger numbers of subjects. If these studies will confirm these new findings, they will probably prompt a change in nomenclature from NCGS to wheat sensitivity to reflect the fact that, beside gluten, other components of wheat may be responsible for the symptoms reported by NCGS patients.
